# Association of adiponectin with hepatic steatosis: a study of 1,349 subjects in a random population sample

**DOI:** 10.1186/1756-0500-7-207

**Published:** 2014-04-03

**Authors:** Marion Flechtner-Mors, Samuel N George, Suemeyra Oeztuerk, Mark M Haenle, Wolfgang Koenig, Armin Imhof, Bernhard O Boehm, Tilmann Graeter, Richard A Mason, Wolfgang Kratzer, Atilla S Akinli

**Affiliations:** 1 Institute of Epidemiology and Medical Biometry, Albert-Einstein-Allee 41, Ulm 89081, Germany; 2 Department of Internal Medicine I, University Hospital Ulm, Albert-Einstein-Allee 23, Ulm 89081, Germany; 3 Department of Internal Medicine II, University Hospital Ulm, Albert-Einstein-Allee 23, Ulm 89081, Germany; 4 Department of Diagnostic and Interventional Radiology, University Medical Center Ulm, Albert-Einstein-Allee 23, Ulm 89081, Germany; 5 Louis Stokes Cleveland Department of Veterans Affairs Medical Center, 10700 East Boulevard, Cleveland, Ohio 44106, USA

**Keywords:** Ultrasonography, Fatty liver, NAFLD, Adipose tissue, Cross-sectional studies

## Abstract

**Background:**

Objective of the present study was to examine the association between adiponectin and hepatic steatosis, and other biochemical and anthropometric parameters in healthy subjects.

**Results:**

A total of 1349 subjects (age 18–65 years) underwent ultrasound examination of the liver. Mean adiponectin concentration for the study collective was 11.35 ± 6.28 μg/mL. The following parameters were assessed for their association with adiponectin: body-mass index (BMI); age; sex; arterial blood pressure; nicotine use; alcohol consumption; physical activity; metabolic syndrome; total, low-density lipoprotein (LDL) and high-density lipoprotein (HDL) cholesterol; triglycerides; aspartate aminotransferase (AST); alanine aminotransferase (ALT); γ-glutamyltransferase (GGT); alkaline phosphatase (AP); C-reactive protein (CRP); insulin sensitivity according to the Homeostasis Model Assessment (HOMA); random blood glucose; and the degree of steatosis of the liver. The numerical differences in the variables influencing adiponectin returned in the descriptive analysis were confirmed at bivariate analysis for BMI, ALT, AST, GGT, AP, total and HDL cholesterol, triglycerides, CRP, arterial blood pressure, metabolic syndrome, nicotine use and alcohol consumption. The logistic regression of the multivariate analysis showed that male sex, hepatic steatosis, BMI, metabolic syndrome, tobacco smoking and CRP correlate negatively with adiponectin, while age, moderate alcohol consumption and HDL cholesterol exhibit a positive association.

**Conclusions:**

The results of the present study confirm the findings of previous research. Adiponectin correlates negatively with cardiometabolic risk factors and is an independent indicator for non-alcoholic fatty liver disease (NAFLD).

## Background

Over the past several years, non-alcoholic fatty liver disease (NAFLD) has gained increasing importance, both as a clinical entity and as a focus of research [1]. NAFLD is characterized by an increased accumulation of fat in the hepatocytes in the absence of excessive alcohol consumption [2]. Its prevalence worldwide is estimated in the range of 20-30%. NAFLD includes both non-alcohol fatty liver (NAFL), characterized by a simple increase in the fat content of the liver, and non-alcoholic steatohepatitis (NASH) with increased fat content and inflammatory infiltrates. About 10-20% of patients with NAFL develop NASH, which may ultimately lead to cirrhosis of the liver and to hepatocellular carcinoma (HCC) [3-5].

While NAFLD is closely associated with obesity, type 2 diabetes mellitus and coronary artery disease (CAD), it may occur in the absence of type 2 diabetes and CAD [6,7]. The increased fat content of the liver correlates positively with the insulin resistance that characterizes metabolic syndrome [8]: hence, NAFLD is often considered an hepatic manifestation of metabolic syndrome [9]. It remains unclear, however, whether insulin resistance triggers an increase in the fat content of the liver or whether an increased fat content of the liver precedes the development of insulin resistance or causes insulin resistance *per se*[10]. It is known that adiponectin, a peptide hormone released from adipose tissue affects both the fat content of the liver and the development of NAFLD [11].

Unlike most adipose tissue hormones, adiponectin shows decreased concentrations in obesity and correlates negatively with both cardiometabolic risk factors and with the fat content of the liver [12,13]. Adiponectin concentrations are 20-60% lower in patients with NAFLD than in healthy persons [8]. There is evidence in the literature that adiponectin at sufficiently high concentrations may protect against the development of NAFL [14].

Objective of the present study was to analyze the association between adiponectin and cardiometabolic risk factors, and with sonographically diagnosed hepatic steatosis in a healthy population of a small German town.

## Results

### Descriptive analysis

The study collective consisted of 1,349 subjects (733 females, 54.3%; 616 males, 45.7%; mean age, 41.3 ± 12.5 years). Among all subjects, 24.7% exhibited sonographic evidence of hepatic steatosis, 47.8% were overweight, 12.2% had a history of hypertension, 3.9% fulfilled the (modified) criteria for metabolic syndrome, 50.1% were current or former smokers, 87.3% reported no or very low consumption of alcohol, and 61.2% described engaging in physical exercise.

The mean plasma concentration of adiponectin in all 1,349 study subjects was 11.35 ± 6.28 μg/mL. The mean adiponectin concentrations of subjects broken down by age, degree of hepatic steatosis, BMI, metabolic syndrome, alcohol consumption and physical exercise are given in Table 1. Table 2 gives mean adiponectin concentrations of subjects broken down according to laboratory parameters that were within, above or below the respective reference ranges.

**Table 1:** The concentration of adiponectin in 1,349 healthy subjects

	**N**	**Adiponectin (μg/ml) Mean ± SD**
**Sex**		
Females	733	13,87 ± 6,77
Males	616	8,34 ± 3,89
**Age (years)**		
18-30	274	10,77 ±,44
31-40	400	10,97 ± 6,08
41-50	312	11,22 ± 6,34
51-65	363	12,30 ± 6,91
**Hepatic steatosis**		
Grad 0	1016	12,02 ± 6,44
Grad 1	153	10,27 ± 6,07
Grad 2/3	180	8,47 ± 4,27
**Body Mass Index (kg/m**^**2**^**)**		
<25	704	12,65 ± 6,64
25-30	437	10,14 ± 5,61
>30	208	9,48 ± 5,30
**Hypertension**		
No	1174	11,48 ± 6,29
Yes	164	10,59 ± 6,18
Unknown	11	8,30 ± 4,96
**Metabolic syndrome**		
No	1296	11,51 ± 6,31
Yes	53	7,38 ± 3,64
**Tobacco smoking**		
No	672	12,16 ± 6,76
Yes	385	10,34 ± 5,85
Former smoker	292	10,81 ± 5,35
**Alcohol**		
Ex-drinker	40	9,78 ± 5,51
0 g	509	11,32 ± 6,20
1-20g/Tag	671	11,89 ± 6,65
21-40g/Tag	129	9,14 ± 3,79
**Physical exercise**		
No	523	11,19 ± 6,52
Yes	826	11,44 ± 6,12

SD = standard deviation.

**Table 2:** Adiponectin concentration broken down according to laboratory parameters

	**N**	**Adiponectin (μg/ml) Mean ± SD**
**ALT**		
Normal	1172	11.68 ± 6.38
Elevated	177	9.13 ± 5.05
**AST**		
Normal	1319	11.36 ± 6.22
Elevated	30	10.55 ± 8.32
**GGT**		
Normal	1196	11.34 ± 6.22
Elevated	114	11.02 ± 7.08
Reduced	39	12.55 ± 5.36
**AP**		
Normal	1167	11.03 ± 6.27
Elevated	7	13.98 ± 7.43
Reduced	175	13.38 ± 5.91
**Triglycerides**		
Normal	952	12.25 ± 6.65
Increased	397	9.19 ± 4.63
**Cholesterol**		
Normal	500	10.98 ± 6.58
Increased	849	11.56 ± 6.09
**HDL-Cholesterol**		
Normal	1050	10.22 ± 5.39
Increased	299	15.32 ± 7.46
**LDL-Cholesterol**		
Normal	538	11.58 ± 6.49
Erhöht	811	11.19 ± 6.13
**Random Glucose**		
Normal	1305	11.32 ± 6.27
Elevated	37	11.70 ± 5.72
Reduced	7	14.21 ± 10.16
**CRP**		
Normal	1192	11.48 ± 6.30
Elevated	157	10.34 ± 6.03
**HOMA**		
Normal	211	10.49 ± 6.46
Elevated	33	9.20 ± 5.66

ALT = Alanine aminotransferase, AP = Alkaline phosphatase, AST = Aspartate aminotransferase, CRP = C-reactive protein, GGT = γ-glutamyl-transferase, HDL = High Density Lipoprotein, HOMA = Homeostasis Model Assessment, LDL = Low Density Lipoprotein Random-Glucose = Glucose independent of meal times; HOMA = Homeostasis Model Assessment, SD = standard deviation.

### Bivariate analysis

There was a statistically significant negative correlation between the adiponectin concentration and BMI. The hepatic function (ALT, AST, GGT) and cholestasis parameters (AP) also correlated negatively with adiponectin. Among the lipids, triglycerides showed a negative correlation, while total cholesterol showed a weak positive correlation and HDL cholesterol a narrowly positive correlation. LDL cholesterol showed a negative correlation without, however, attaining statistical significance. Random blood glucose and insulin sensitivity (HOMA) also showed a negative correlation with adiponectin concentrations, though likewise failing to achieve statistical significance (Table 3).

**Table 3:** Correlation coefficient (r) between the adiponectin concentration and different laboratory parameters, and BMI

	**r**	**p**
**ALT**	- 0.325	<0.0001
**AST**	- 0.173	<0.0001
**GGT**	- 0.311	<0.0001
**AP**	- 0.176	<0.0001
**Cholesterol**	0.062	0.0235
**LDL-Cholesterol**	- 0.050	0.0675
**HDL-Cholesterol**	0.521	<0.0001
**Triglycerides**	- 0.310	<0.0001
**Random blood glucose**	- 0.040	0.1390
**HOMA**	- 0.103	0.1080
**CRP**	- 0.068	0.0128
**BMI**	- 0.292	<0.0001

ALT = Alanine aminotransferase, AP = Alkaline phosphatase, AST = Aspartate aminotransferase, BMI = Body-Mass Index, CRP = C-Reactive Protein, GGT = γ-glutamyl-transferase, HDL = High Density Lipoprotein, HOMA = Homeostasis Model Assessment, LDL = Low Density Lipoprotein Random-Glucose = Glucose independent of meal times; HOMA = Homeostasis Model Assessment, SD = standard deviation; Random glucose = Glucose independent of time of day.

Hypertension, metabolic syndrome, hepatic steatosis and tobacco use showed a negative correlation with adiponectin (*p* < 0.0001). There was a positive correlation with age and alcohol consumption (*p* < 0.0001). There was no clear correlation between physical exercise and the adiponectin concentration (*p* < 0.3285).

### Multivariate analysis

Multivariate analysis demonstrated a negative correlation between the adiponectin concentration and the factors male sex, hepatic steatosis, BMI, metabolic syndrome and tobacco smoking. By comparison, age correlated positively with the adiponectin concentration. While HDL-cholesterol exhibited a positive correlation with adiponectin, the correlation with CRP was negative (Table 4).

**Table 4:** Association of the adiponectin concentration with various parameters in the multivariate regression analysis

	**b**	**p**
**Male sex**	- 0.318	<.0001
**Hepatic steatosis**	- 0.064	0.0475
**BMI**	- 0.010	0.0011
**Age**	0.004	<.0001
**Triglycerides**	- 0.024	0.1242
**HDL Cholesterol**	0.356	<.0001
**CRP**	- 0.004	0.0434
**Metabolic syndrome**	- 0.128	0.0434
**Tobacco smoking**	- 0.065	0.0117
**Alcohol consumption (0-20 g/d)**	0.052	0.0327
**Alcohol consumption (20-40 g/d)**	0.093	0.0380

BMI = Body-mass index, CRP = C-reaktives Protein, HDL = High-Density-Lipoprotein.

## Discussion

The present study examined the largest collective of healthy subjects assessed to date for an association between sonographically diagnosed hepatic steatosis and plasma adiponectin concentrations. Sonographic evidence of hepatic steatosis was documented in 24.7% of subjects. The multivariate analysis of our data shows a negative correlation between adiponectin and hepatic steatosis. Breaking down study subjects according to sonographically determined hepatic fat content into groups with grade 0, 1 and 2/3 steatosis, it was observed that adiponectin concentrations decline inversely to the degree of steatosis.

This observation corresponds to findings of Pisto et al. in which an increase in hepatic fat content, estimated by sonographic assessment of brightness into levels 0, 1 and 2, was associated with a decrease in adiponectin concentrations [15]. The results of that study and ours are in good agreement even though the earlier study measured comparatively higher adiponectin concentrations. These differences in adiponectin concentrations may possibly be due to the different age distribution of the two study collectives as since the concentration of adiponectin in the blood increases with age [16].

In our study collective, the mean adiponectin concentration was significantly higher in women than in men. This known difference between the sexes emerges at puberty and efforts have been made to explain it on the basis of differences in androgen levels [16,17]. Experimental studies, however, have failed to demonstrate a direct influence of androgens on adiponectin concentrations. It has been postulated that the sex hormones regulate as yet unknown factors that, in turn, affect the adiponectin concentration [18].

In our study collective, BMI correlates negatively with the adiponectin concentration. These results correspond with those of earlier studies [19]. However, it has also been shown that adiponectin is not associated with the BMI or subcutaneous adipose tissue but correlates significantly and negatively with the mass of visceral adipose tissue [20]. It was furthermore shown that adiponectin concentrations correlated negatively with the mass of visceral fat but positively with the mass of subcutaneous fat [21]. Contradictory study findings regarding the association between adiponectin and BMI are possibly the result of the different composition of the respective study collectives with respect to the degree of overweight and the relative proportions of subcutaneous and visceral adipose tissue [21].

A total of 61.2% of the subjects in our study population were physically active (by self-report). Those who engaged in physical exercise showed higher mean adiponectin concentrations than did subjects who did not report being physically active. Multivariate analysis, however, failed to demonstrate a statistically significant difference in adiponectin levels between these groups. It is possible that the intensity of physical exercise was too low or the difference in physical activity between the groups was too small. A review of the literature reveals that participation in a physical exercise program is associated with an increase in adiponectin concentrations in the blood [22,23] and that the fat content and histological signs of inflammation in the liver are reduced [24]. The association between adiponectin and cardiovascular risk factors and NAFLD were described. There was a correlation between adiponectin and steatosis hepatis, and there was a correlation between adiponectin and hypertension. The association between fatty liver and hypertension is well known [25]. Further NAFLD with or without increased ALT levels is associated with progression of blood pressure over time and incident hypertension [26,27]. In our study out of 1349 healthy subjects only 80 subjects had NAFLD. Because of the low portion of subjects with hypertension the relationship between hypertension and hepatic steatosis was not analyzed in detail.

In our study, adiponectin concentrations correlated negatively with triglycerides and positively with HDL-cholesterol. These observations correspond to the findings of other studies [28]. The action of adiponectin on triglyceride concentrations is ascribed to an increase in fatty acid oxidation via sequential activation of AMPK, p38 MAPK and PPAR-α [29]. A direct action of adiponectin on the catabolism of apoprotein A-I explains the positive correlation between adiponectin and HDL-cholesterol [30].

Only 3.9% of subjects participating in the present study fulfilled the minimum number of criteria required for diagnosing metabolic syndrome. The low prevalence of metabolic syndrome is most likely due to the fact that diabetics, who very frequently suffer from metabolic syndrome, were not included in the study [31].

Adiponectin was negatively associated with plasma CRP concentrations. This association confirms observations reported by other authors [28]. Anti-inflammatory properties have also been described for adiponectin and this may be important in the progression of NAFL to NASH [32,33]. Evidence from animal experiments suggests that adiponectin attenuates signs of inflammation and may protect against the development of NASH [34]. It has recently been shown in one prospective study and in another longitudinal study that a high adiponectin concentration protects against development of metabolic syndrome [35,36].

In the present study, multivariate analysis showed a significant positive association between adiponectin and moderate alcohol consumption. Earlier randomized cross-over and intervention studies had also reported that moderate alcohol consumption was associated with an increase in adiponectin levels [37,38]. In contrast to the findings of studies conducted in Europe, cross-sectional studies from Japan found a negative association between adiponectin and alcohol consumption [39]. These divergent findings may have an ethnic basis or related to subjects’ environment.

In our study, adiponectin concentrations were lower in those subjects classified as tobacco smokers than in non-smokers. This corresponds to the finding that, in non-smokers, acute exposition to tobacco smoke results in a decline in adiponectin levels within 12 hours and that smokers who abstain from smoking exhibit an increase in adiponectin concentrations within two months [40,41]. The reduced adiponectin level in smokers may be explained by an inhibitory effect of nicotine on the expression of adiponectin from fat cells [40]. The role of genetic background in the association between adiponectin and fatty liver was not addressed in this study. However, it is known from the literature that adiponectin gene rs266729 polymorphism increases the risk of NAFLD [42]. Also the transcription factor sterol regulatory element-binding protein-1 c (SREBP-1c) may contribute to the development of NAFLD. The SREBP-1c gene rs11868035 polymorphism is associated with a high risk of NAFLD [43]. Further genetic variants in the Patatin like phospholipase-3 (PNPLA3) gene are associated with increased risk of nonalcoholic fatty liver disease [44,45]. PNPLA3 rs738409 (I148M) genotypes may represent a genetic determinant of serum adiponectin concentrations and may be involved in mediating the susceptibility of liver steatosis [46].

Hypertension and NAFLD both are components of the metabolic syndrome and are associated with low levels of adiponectin. Increased expression of adiponectin or administration of recombinant adiponectin corrects hypertension and NAFLD. This implies a therapeutic potential of adiponectin [47]. Further low levels of adiponectin are found in subjects with diabetes and coronary artery disease [48]. Raising adiponectin levels or enhancing activity of adiponectin receptors may attenuate the components of the metabolic syndrome and may improve the management of diabetes and of cardiovascular diseases [49].

The main strength of the present study is that it examined a healthy population under normal domestic conditions using standardized methods. One weakness is that only 60% of the selected population actually participated in the study. This is due in part to the exclusion of subjects with liver diseases and diabetes mellitus, as well as subjects with incomplete documentation. Other drawbacks were that the study is a cross-sectional study with singular observations and that collection of personal data were based on questionnaires. The study was aimed at the association between adiponectin and NAFLD. In the population the portion of participants with hypertension and metabolic syndrome were relatively low. Larger studies with patients with the metabolic syndrome or with hypertension are necessary to confirm the observations made in this population based study. Another weakness of the present study is that the genetic background of the association of adiponectin with steatosis and hypertension is not addressed. The relationship between normotensive and hypertensive blood pressure values and adiponectin concentrations was established in our study. Previously, the glycoprotein PI(A2) allele was identified as an risk factor for stroke in high-risk hypertensive patients [50]. In the present study, we did not investigate glycoprotein IIIa PI(A1/A2) polymorphism in subjects with hypertension. It is possible that the PI(A1/A2) polymorphism is important for development of both hypertension and NAFLD. In patients with CAD, for instance, the presence of the PIA2 allele was associated with a higher risk of cardiovascular events, while, in hypertensive patients with cerebrovascular events, the PIA2 allele increased the risk of stroke [51]. As hypertension is the most frequent component of the metabolic syndrome, the genetics underlying the regulation of blood pressure may be crucial for the development of cardiovascular and cerebrovascular events. Understanding the underlying pathogenetic mechanisms of hypertension may lead to more effective strategies of treatment. Recently, it has been shown that the CaMKIIV gene is involved in regulation of vascular tone and blood pressure. In hypertensive patients, a CaMKIV polymorphism identifies a subset of patients with higher blood pressure values [52]. It may be assumed that the CaMKIV gene deletion not only induces hypertension and cardiovascular disease but also NAFLD. Further limitations of the study include the fact that liver biopsies were not performed to confirm the sonographic diagnosis of NAFLD and data on alcohol and nicotine use, as well as physical activity, were self-reported by study subjects. In addition, for logistical reasons, blood samples were obtained throughout the day meaning that dietary parameters should be considered critically. In this respect limitation is related to the definition of the metabolic syndrome, that divergent from the NCEP ATP III definition, random blood glucose instead of fasting blood glucose was used as one out of three criteria required.

## Conclusion

The present study investigated factors associated with adiponectin in a collective of healthy subjects examined by means of diagnostic ultrasound.

The numerical differences in the variables influencing adiponectin returned in the descriptive analysis were confirmed at bivariate analysis for BMI, ALT, AST, GGT, AP, total and HDL cholesterol, triglycerides, CRP, arterial blood pressure, metabolic syndrome, nicotine use and alcohol consumption.

Male sex, BMI, triglycerides, CRP, tobacco smoking, metabolic syndrome and non-alcoholic fatty liver correlate negatively with adiponectin concentrations. There was a positive association between adiponectin concentrations and age, moderate alcohol consumption and plasma HDL-cholesterol levels. These findings underscore the importance of adiponectin as a biomarker for cardiometabolic risk factors and as an indicator for the severity of non-alcoholic fatty liver.

## Methods

### Collective

**Figure 1:**
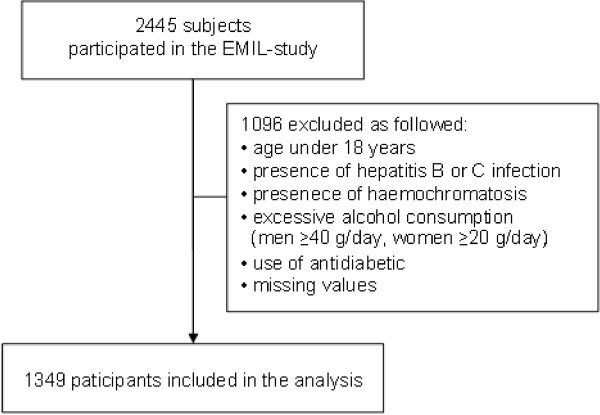
Flow of the subjects across the study.

Four-thousand persons were selected randomly from the registered population of a southern German city (total population, 12,475). Of these, a total of 2,445 persons participated in the study. Subjects completed a standardized questionnaire regarding personal data, lifestyle habits and medical history. Subjects underwent ultrasound examination to determine the fat content of the liver [53]. Subjects with history of liver diseases, those treated with antidiabetic agents, and those with incomplete data were excluded from the study (Figure 1). Of the remaining 1,349 subjects, 54.3% were women.

The study was conducted in conformity with the principles of the Helsinki Declaration and Good Clinical Practice recommendations and was approved by the Ethics Commission of the State Medical Council of Baden-Württemberg (No. 133–02, 24 September 2002). All study participants provided their informed written consent.

### Questionnaire

Each subject received a questionnaire that was completed under the supervision of a trained interviewer. Subjects provided information on their date of birth, sex, body height, body weight, social history, medical history, alcohol consumption, smoking habits and physical activity. In order to validate the results multiple crosschecked questions on the same topic were addressed to the participants. The interview was partially based on validated instruments from other, predominantly cardiovascular, studies (i. e. alcohol questions from the MONICA Study). Further details on the questionnaire has been published in a previous work of the EMIL-study group [53].

### Laboratory studies

Blood samples for laboratory examinations were obtained by means of phlebotomy of a cubital vein. Routine laboratory testing was performed in the Department of Clinical Chemistry of the University Hospital of Ulm using the Dimension XL unit (Dade Behring Inc, Newark, DE, USA) in accordance with guidelines of the International Federation of Clinical Chemistry and Laboratory Medicine (IFCC). Adiponectin concentrations were determined radioimmunologically using the Human Adiponectin RIA Kit (Catalog HADP-61HK, Biotrend Chemikalien GmbH, Cologne, Germany). LDL cholesterol concentrations were calculated using the Friedewald formula. The diagnosis of metabolic syndrome was made in accordance with the National Cholesterol Education Program Adult Treatment Panel III criteria (NCEP-ATP III) [54] with the exception that random blood glucose levels were substituted for the fasting glucose concentration required by NCEP-ATP III.

### Ultrasound

All subjects underwent ultrasound examination of the liver. Examinations were performed by specially trained examiners using four identical HDI 5000 ultrasound scanners (ATL Ultrasound, Philips Medical Systems, Bothell, WA, USA) under the supervision of an experienced sonographer. Findings were documented using a standardized recording form. Based on a sonographic comparison of the hepatic and renal parenchyma, assessment of dorsal echo attenuation by the liver, the visualization of the diaphragm and the hepatic vessels, the degree of hepatic steatosis was classified as “none” (grade 0), or as “mild” (grade 1), “moderate” (grade 2), or “severe” (grade 3) fatty liver disease [55]. The sonographic grading of hepatic steatosis into severity grades 0 to 3 has been shown to be clinically reliable and useful [56].

### Statistical analysis

Statistical calculations were performed using the SAS statistical software package (version 9.2; SAS Institute, Cary, NC, USA). Constant laboratory values were divided into classes with respect to their reference ranges. Historical data were divided according to reasonable criteria. Data were first analyzed descriptively. Categorical variables were represented with absolute and relative frequencies. Mean and standard deviation were determined for constant variables.

The correlation of adiponectin with quantitative variables was calculated using the Spearman rank correlation. The Wilcoxon rank sum test or the Kruskal-Willis test were used for qualitative variables. Multivariate linear regression was used to test the influence of individual parameters on adiponectin concentrations. Adiponectin concentrations were log transformed in order to achieve a normal distribution. All tests were two-tailed. Statistical significance was established at α = 5%.

## Abbreviations

ALT: Alanine aminotransferase; AMPK: Activated protein kinase; AP: Alkaline phosphatase; AST: Aspartate aminotransferase; BMI: Body-mass index; CAD: Coronary artery disease; CRP: C-reactive protein; GGT: γ-glutamyltransferase; HCC: Hepatocellular carcinoma; HDL: High-density lipoprotein; HOMA: Homeostatic model assessment; LDL: Low-density lipoprotein; MAPK: Mitogen-activated protein kinase; NAFL: Non-alcohol fatty liver; NAFLD: Non-alcoholic fatty liver disease; NASH: Non-alcoholic steatohepatitis; NCEP-ATP III: National cholesterol education program adult treatment panel III; PPAR: Peroxisome proliferator-activated receptor; SD: Standard deviation.

## Competing interests

The authors declare that they have no competing interests.

## Authors’ contribution

MFM, SG, SO, MMH, WKoe, AI, BOB, WK and ASA were involved in the design and conduct of the study. SG, SO, MMH, WKoe, AI, BOB, and TG collected and analysed the data. MFM, SG, SO, MMH, RAM, WK and ASA were involved in data interpretation and manuscript writing. All authors read and approved the final version.
